# Smoking and risk of cholangiocarcinoma: a systematic review and meta-analysis

**DOI:** 10.18632/oncotarget.20141

**Published:** 2017-08-10

**Authors:** Yuenan Huang, Liuping You, Weimin Xie, Li Ning, Jinghe Lang

**Affiliations:** ^1^ Department of General Surgery, The Second Affiliated Hospital, Harbin Medical University, Harbin, China; ^2^ Department of Obstetrics and Gynecology, Peking Union Medical College Hospital, Chinese Academy of Medical Sciences and Peking Union Medical College, Beijing, China

**Keywords:** smoking, cholangiocarcinoma, risk, meta-analysis

## Abstract

Previous studies evaluating the association between smoking and risk of cholangiocarcinoma (CCA) have yielded controversial results. We conducted a meta-analysis to evaluate the association based on available evidence. We searched the databases of Embase, PubMed and Cochrane Central Register of Controlled Trials from inception to April 11, 2017. Studies that investigated the association between smoking and risk of CCA were included. Pooled odds ratio (OR) estimates and 95% confidence intervals (CIs) were calculated using either a random-effects or a fixed-effects model. A total of 22 studies involving 324,333 participants were identified. The summary OR of CCA was 1.31 (95% CI, 1.15 to 1.51) for smokers versus nonsmokers. The increased risk was independent of diabetes mellitus, bilious tract stone disease, and liver cirrhosis. Smokers also had increased risk of intrahepatic CCA (12 studies; OR, 1.31; 95% CI, 1.06 to 1.63) and extrahepatic CCA (12 studies; OR, 1.32; 95% CI, 1.10 to 1.59) compared with nonsmokers. The results of our meta-analysis support the hypothesis that there is a moderate association between cigarette smoking and risk of CCA.

## INTRODUCTION

Cholangiocarcinoma (CCA), the second most common primary hepatobiliary carcinoma, is an enigmatic and aggressive malignancy originating from the epithelium of the biliary tract system [1]. It can be classified into two anatomic subtypes: intrahepatic and extrahepatic [2]. Extrahepatic CCA can be further divided anatomically into perihilar and distal CCA [3]. CCA is a devastating carcinoma with a dismal 5-year overall survival rate of less than 10% [4]. Despite the significant geographic variation in its incidence, worldwide epidemiological data have shown an increasing trend in the past few years [5]. Several risk factors have been proposed to be involved in the etiology of CCA. Certain diseases, including primary sclerosing cholangitis, cholelithiasis, hepatitis B and C infection, and diabetes mellitus, have been linked to increased risk of CCA [2, 6–9]. Parasite infection with *Opisthorchis viverrini* and *Clonorchis sinensis*, and exposure to the toxic agent thorotrast have also been associated with the development of CCA [10–11]. However, these risk factors are quite variable in different areas of the world, and some remain controversial.

Several compounds of cigarette smoke have been shown to have a carcinogenic effect in preclinical studies. For instance, N-Nitrosodimethylamine is carcinogenic in many species including rats, mice and monkeys, and is known to cause liver cancer [12]. Interestingly, it has also been shown to cause CCA in mice [13]. Cigarette smoking has been shown to have a tumorigenic effect in a wide variety of malignancies, including the oral cavity, pharynx, larynx, lung, esophagus, stomach, pancreas, liver, cervix, and kidney [14]. As such, smoking is strongly associated with malignancies of the respiratory tract system, but even the gastrointestinal and urogenital systems show a substantial increase in cancer risk with smoking. However, the evidence for CCA is contradictory.

This study aimed to evaluate the association between smoking and risk of CCA through a systematic review of available evidence.

## RESULTS

### Study selection

**Figure 1:**
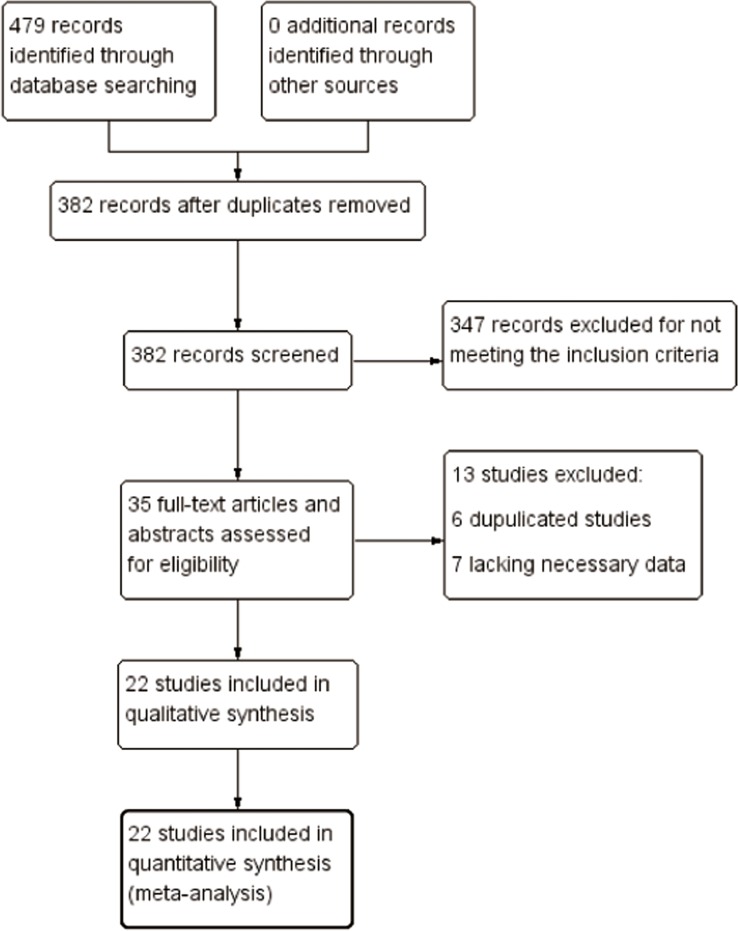
Study flow diagram

A total of 479 records were identified through database searching; no additional records were found from ongoing trials and conference proceedings. After removing duplicates and strict screening, 35 potentially relevant records were retrieved for detailed review. A total of 13 of these 35 records were subsequently excluded for the following reasons: six were duplicate reports from the same population [15–20], and seven did not have data specific for CCA [21–27]. No records were identified from reference lists. Thus, a total of 22 studies were included in this meta-analysis [28–49]. The flow diagram summarizing the selection process is given in Figure 1.

### Study characteristics

All included studies were case controls [28–49], that included a total of 324,333 participants and published between 1993 and 2016. The number of patients with CCA ranged from 6 to 2,395. One of the 22 studies was published only in abstract form [47]. Eight studies were carried out in North America [28, 29, 31, 32, 35, 36, 43, 49], 4 in Europe [33, 39, 44, 46], and 10 in Asia [30, 34, 37, 38, 40–42, 45, 47, 48]. The Newcastle–Ottawa scale (NOS) values ranged from four to eight stars; 12 of the 22 studies were of high quality [29–31, 35, 37, 40, 42, 44–46, 48, 49]. The characteristics of the included studies are shown in Table 1.

**Table 1:** Characteristics of included case-control studies

First author	Country	Type of carcinoma	Cases (*N*)	Contrls (*N*)	Study period	Study population	Definition of smoking (ever, current)	Adjustment factors^*^	NOS value
Ghadirian et al. 1993 [28]	Canada	CCA	24	239	1984–1988	Men and women	Ever: NR	1–6	6
Chow et al. 1994 [29]	USA	eCCA	62	248	1985–1989	Men and women	Ever: NR	1, 2	8
Shin et al. 1996 [30]	Korea	iCCA	41	406	1990–1993	Men and women	Ever: Heavy smoking: > 1 pack per day for > 10 years	1, 2, 7	8
Khan et al. 1999 [31]	USA	eCCA	31	138	1980–1994	Men and women	Ever: NR	1, 2, 7–9	8
Chalasani et al. 2000 [32]	USA	CCA	26	87	1991–1998	Men and women with primary sclerosing cholangitis	Ever: NR	10, 11	4
Kuper et al. 2001 [33]	Greece	CCA	6	360	1995–1998	Men and women	Ever: NR	1, 2	5
Yamamoto et al. 2004 [34]	Japan	iCCA	50	205	1991–2002	Men and women	Ever: NR	2, 12	6
Shaib et al. 2007 [35]	USA	iCCA + eCCA	246	236	1992–2002	Men and women	Ever: NR	1, 2, 8	8
Welzel et al. 2007 [36]	USA	iCCA + eCCA	1,084	102,782	1993–1999	Men and women (≥ 65y)	Ever: NR	1, 2, 8, 11, 13	6
Lee et al. 2008 [37]	Korea	iCCA	622	2,488	2000–2004	Men and women	Ever: Subjects were considered smokers if they had smoked for any time before admission	1, 2, 14	8
Zhou et al. 2009 [38]	China	iCCA	317	634	2003–2006	Men and women	Ever: NR	1, 2, 4, 8, 9, 11, 15–21	6
Grainge et al. 2009 [39]	UK	CCA	372	5,760	1987–2002	Men and women	Current: NR	1, 2, 22	5
Tao et al. 2010 [40]	China	iCCA + eCCA	190	380	1998–2008	Men and women	Ever: NR	1, 2	8
Cai et al. 2011 [41]	China	hCCA	313	608	2000–2005	Men and women	Ever: NR	1, 2, 23	6
Liu et al. 2011 [42]	China	iCCA	87	228	2000–2008	Men and women with hepatolithiasis	Ever: A smoker was defined as someone who had smoked 20 cigarettes or more per day for more than 1 year	1, 2, 9, 11, 14, 24, 25	8
Welzel et al. 2011 [43]	USA	iCCA	743	195,953	1993–2005	Men and women (≥ 65y)	Ever: NR	1, 2, 8, 11, 26	6
Onal et al. 2012 [44]	Turkey	iCCA + eCCA	99	48	2006–2010	Men and women	Ever: Ever having smoked cigarettes was defined as having smoked cigarettes ≥ 6 d/wk for ≥ 6 mo.	1, 2	7
Zhou et al. 2013 [45]	China	eCCA	239	478	1999–2011	Men and women	Ever: NR	1, 2, 9, 16, 20, 24, 27	7
Brandi et al. 2013 [46]	Italy	iCCA + eCCA	100	361	2006–2010	Men and women	Ever: NR	1, 2, 11	7
Hosono et al. 2014 [47]	Japan	eCCA	88	547	2009–2013	Men and women	Ever: NR	1, 2	–
Lee et al. 2015 [48]	Korea	pCCA	81	162	2007–2013	Men and women	Ever: NR	1, 2, 9, 14, 20	7
Choi et al. 2016 [49]	USA	iCCA + pCCA + dCCA	2,395	4,769	2000–2014	Men and women	Ever: Ever-smoker was defined as any person having a history of smoking	1, 2, 8, 10, 16, 20, 28–35	8

Abbreviations: CCA: cholangiocarcinoma; iCCA: intrahepatic cholangiocarcinoma; eCCA: extrahepatic cholangiocarcinoma; hCCA: hilar cholangiocarcinoma; pCCA: perihilar cholangiocarcinoma; dCCA: distal cholangiocarcinoma; NOS, Newcastle-Ottawa scale; NR: not reported (number of cigarettes and period of smoking).

^*^1, Age; 2, sex; 3, other smoking habits; 4, alcohol consumption; 5, schooling; 6, respondent status; 7, socioeconomic status; 8, ethnicity; 9, cholelithiasis; 10, primary sclerosing cholangitis; 11, geographic location; 12, operation date; 13, state buy-in status; 14, date of admission or diagnosis; 15, HBV; 16, liver cirrhosis; 17, hepatic cyst; 18, hepatic hemangioma; 19, fatty liver; 20, diabetes mellitus; 21, hepatic schistosomiasis; 22, general practice group; 23, the year of search; 24, family history of cancer,; 25, appendectomy during childhood; 26, Medicare/Medicaid Dual Enrollment; 27, history of cholecystectomy; 28, obesity; 29, hypertension; 30, cerebrovascular accident; 31, coronary artery disease; 32, peripheral vascular disease; 33, atrial fibrillation; 34, nonalcoholic fatty liver disease/nonalcoholic steatohepatitis; 35, inflammatory bowel disease.

### Meta-analysis

#### Smoking and risk of CCA

**Figure 2:**
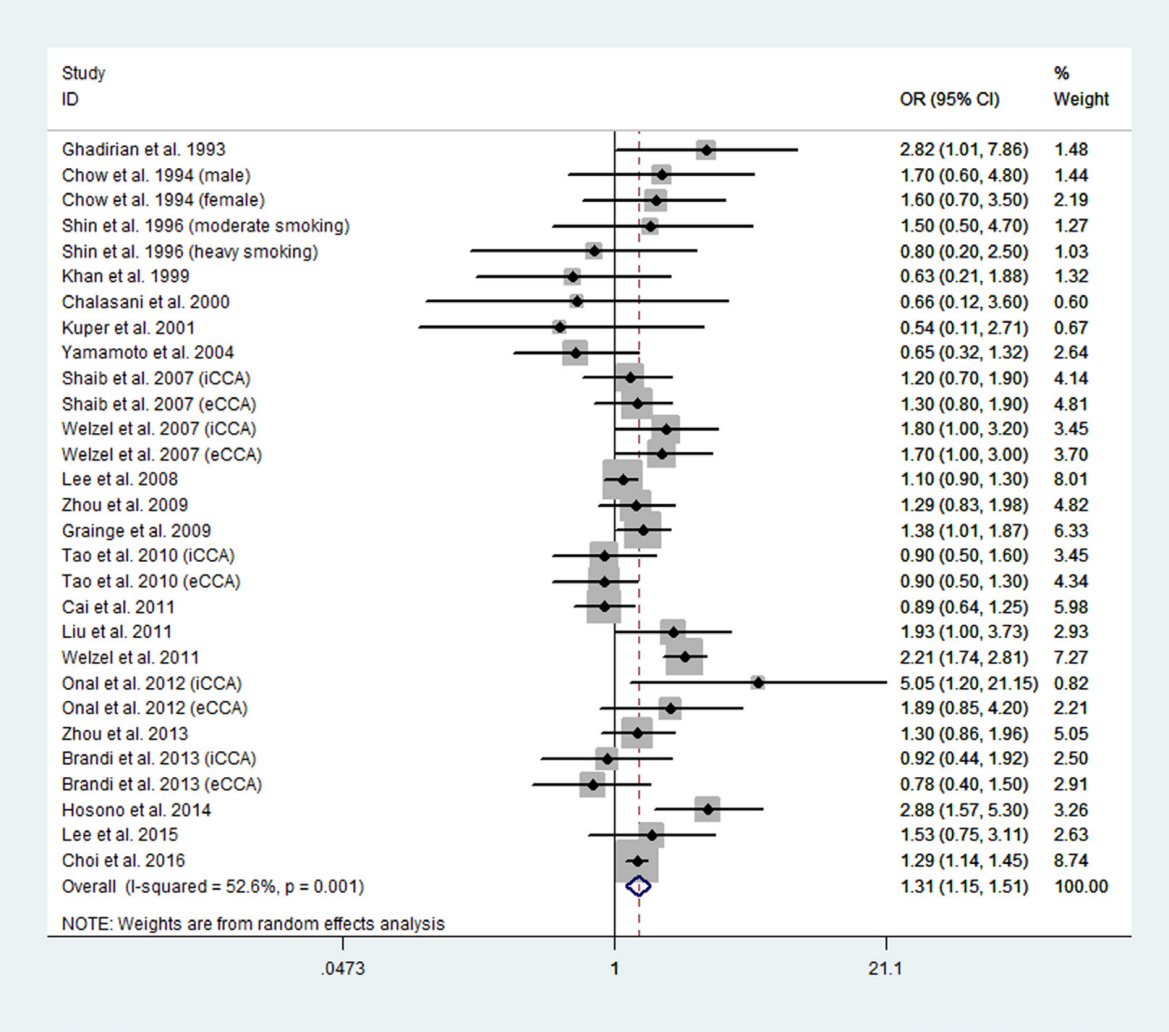
Forest plot of smoking and risk of CCA

A total of 22 case-control studies involving 7,216 CCA cases and 317,117 control cases were analyzed [28–49]. Significant heterogeneity existed among the studies (*P* = 0.001; I^2^ = 52.6%). The summary odds ratio (OR) of CCA was 1.31 [95% confidence interval (CI), 1.15 to 1.51] in the random-effects model for smokers versus nonsmokers (Figure 2). Among these studies, only one study investigated the association between current smoking and risk of CCA, and the results indicated an increased risk of CCA among current smokers (OR, 1.38; 95% CI, 1.01 to 1.87) [39]. Upon evaluating the 21 studies with data for ever smoker only, the pooled data showed that the association between smoking and risk of CCA was similar (OR, 1.31; 95% CI, 1.13 to 1.51). The association between current smokers and ever smokers with the risk of CCA was not different.

Subgroup meta-analyses by study location, study quality, and adjustment for confounders were then performed (Table 2). A significant positive association existed between smoking and risk of CCA in both high-quality studies (OR, 1.23; 95% CI, 1.13 to 1.34) and low-quality studies (OR, 1.35; 95% CI, 1.00 to 1.83). A positive association between smoking and risk of CCA was observed in studies performed in Western countries (OR, 1.44; 95% CI, 1.20 to 1.73), and a positive, but not significant association was found in studies performed in Asian countries (OR, 1.17; 95% CI, 0.97 to 1.42). When we limited the meta-analysis to studies that adjusted for potential confounders, the pooled data also showed an increased risk of developing CCA in smokers in the studies that adjusted for diabetes mellitus (OR, 1.30; 95% CI, 1.16 to 1.45), cholelithiasis (OR, 1.35; 95% CI, 1.05 to 1.72) , and liver cirrhosis (OR, 1.29; 95% CI, 1.15 to 1.44).

**Table 2:** Subgroup analysis

Subgroups	No. of studies	OR (95% CI)	Heterogeneity	
			I^2^ (%)	*P* value
Risk of CCA	22	1.31(1.15 to 1.51)	52.6%	0.001
Study location				
Western	12	1.44 (1.20 to 1.73)	49.1	0.012
Asian	10	1.17 (0.97 to 1.42)	43.6	0.053
Study quality				
High	12	1.23 (1.13 to 1.34)	0.6	0.447
Low	9	1.35 (1.00 to 1.83)	70	0.000
Adjustment for confounders				
Adjustment for diabetes	4	1.30 (1.16 to 1.45)	0.0	0.975
Adjustment for cholelithiasis	5	1.35 (1.05 to 1.72)	0.0	0.526
Adjustment for liver cirrhosis	3	1.29 (1.15 to 1.44)	0.0	0.999
Risk of intrahepatic CCA	12	1.31 (1.06 to 1.63)	66.2	0.000
Study location				
Western	6	1.54 (1.08 to 2.19)	77.0	0.001
Asian	6	1.11 (0.96 to 1.30)	6.2	0.380
Study quality				
High	8	1.17 (1.05 to 1.31)	6.3	0.383
Low	4	1.44 (0.90 to 2.32)	77.1	0.004
Adjustment for confounders				
Adjustment for diabetes	2	1.22 (1.04 to 1.43)	0.0	0.797
Adjustment for cholelithiasis	2	1.45 (1.01 to 2.08)	2.4	0.311
Adjustment for liver cirrhosis	2	1.22 (1.04 to 1.43)	0.0	0.797
Risk of extrahepatic CCA^*^	12	1.32 (1.10 to 1.59)	45.1	0.034
Study location				
Western	7	1.35 (1.17 to 1.55)	15.8	0.301
Asian	5	1.29 (0.88 to 1.90)	69.1	0.012
Study quality				
High	9	1.29 (1.13 to 1.47)	11.3	0.336
Low	2	1.18 (0.63 to 2.22)	74.3	0.049
Adjustment for confounders				
Adjustment for diabetes	3	1.36 (1.16 to 1.58)	11.8	0.334
Adjustment for cholelithiasis	3	1.26 (0.90 to 1.77)	0.0	0.397
Adjustment for liver cirrhosis	2	1.35 (1.15 to 1.58)	39.2	0.193

Abbreviations: CCA, cholangiocarcinoma; OR, odds ratio.

^*^Extrahepatic CCA includes extrahepatic CCA, hilar CCA, perihilar CCA and distal CCA that presented in the studies.

### Smoking and risk of intrahepatic CCA

**Figure 3:**
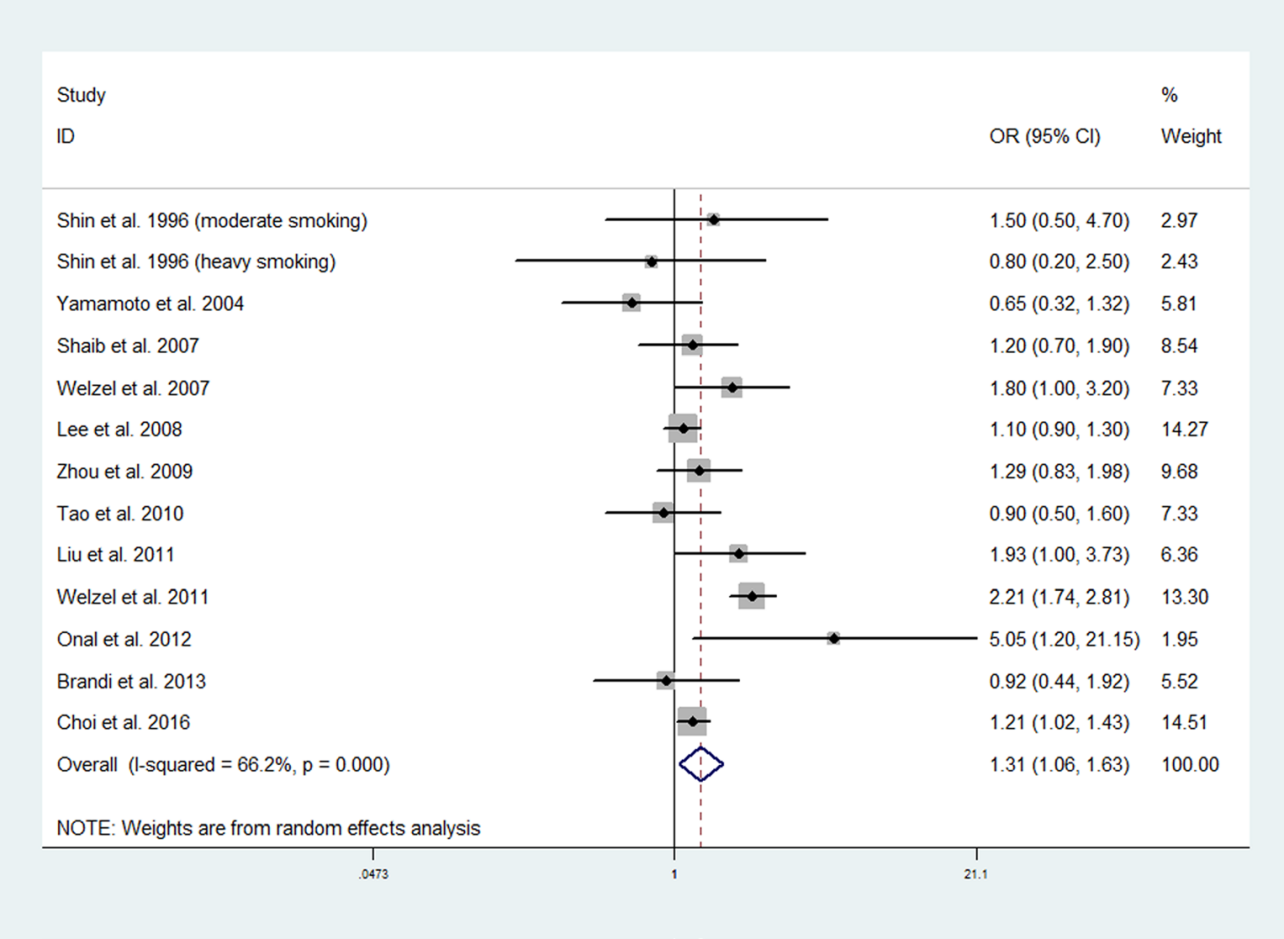
Forest plot of smoking and risk of intrahepatic CCA

Twelve studies involving 3,759 patients with intrahepatic CCA and 308,278 healthy controls investigated the association between smoking and risk of intrahepatic CCA [30, 34–38, 40, 42–44, 46, 49]. A significant heterogeneity existed among the studies (*P* = 0.000; I^2^ = 66.2%). The pooled data using the random effects model showed an increased OR of developing intrahepatic CCA in smokers (OR, 1.31; 95% CI, 1.06 to 1.63) (Figure 3).

In the subgroup meta-analyses, a positive significant association was found between smoking and risk of intrahepatic CCA in western studies (OR, 1.54; 95% CI, 1.08 to 2.19) and high-quality studies (OR, 1.17; 95% CI, 1.05 to 1.31). The association between smoking and risk of intrahepatic CCA was positive, but not significant in studies performed in Asian countries (OR, 1.11; 95% CI, 0.96 to 1.30) and low quality studies (OR, 1.44; 95% CI, 0.90 to 2.32). When the meta-analysis was limited to studies that adjusted for potential confounders, the pooled data showed an increased risk of developing CCA in smokers in the studies that adjusted for diabetes mellitus (OR, 1.22; 95% CI, 1.04 to 1.43), cholelithiasis (OR, 1.45; 95% CI, 1.01 to 2.08) , and liver cirrhosis (OR, 1.22; 95% CI, 1.04 to 1.43).

### Smoking and risk of extrahepatic CCA

**Figure 4:**
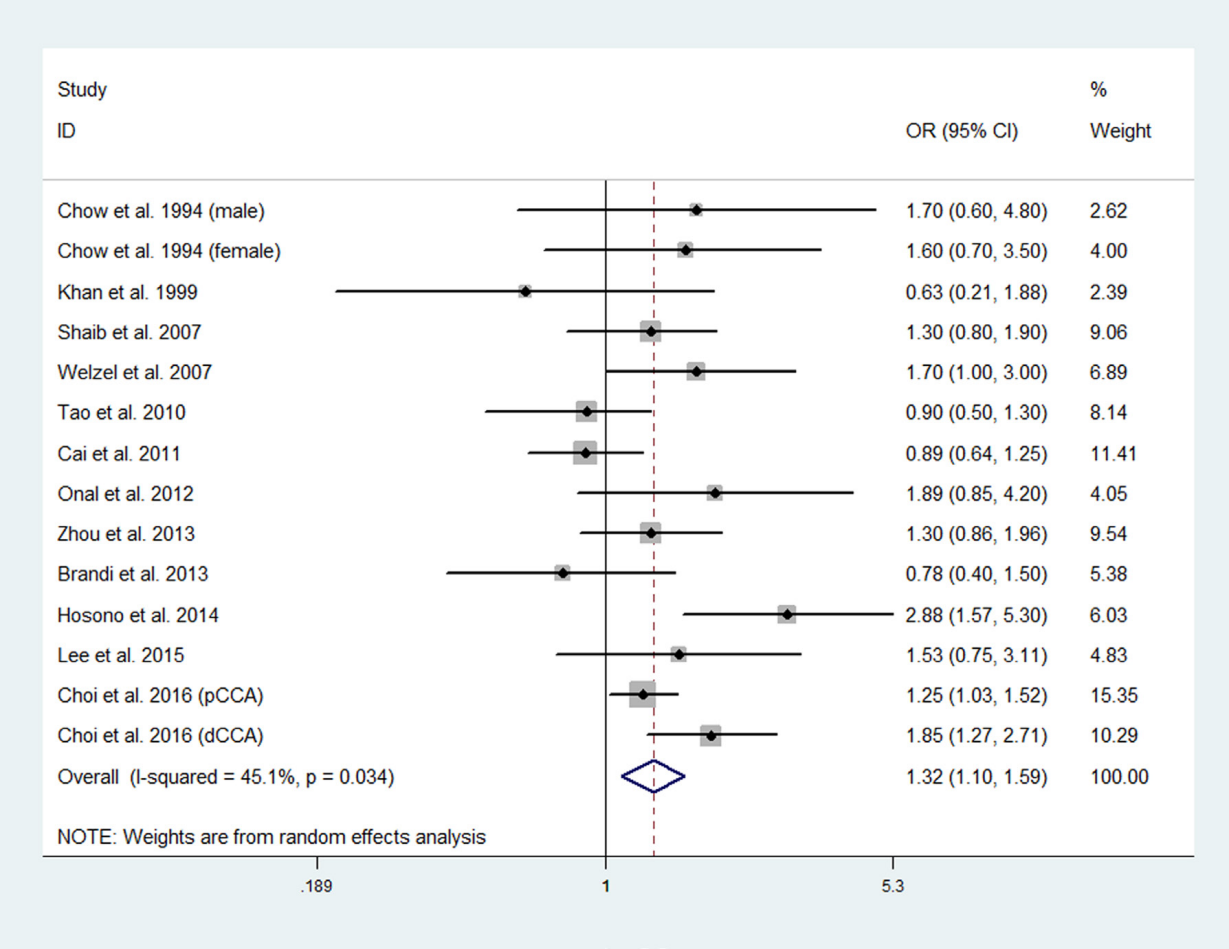
Forest plot of smoking and risk of extrahepatic CCA

Twelve studies involving 3029 patients with extrahepatic CCA and 110,608 healthy controls explored the association between smoking and risk of extrahepatic CCA [29, 31, 35, 36, 40, 41, 44–49]. A significant heterogeneity existed among the studies (*P* = 0.034; I^2^ = 45.1%). The pooled data using the random-effects model showed that smoking was associated with improved risk of extrahepatic CCA (OR, 1.32; 95% CI, 1.10 to 1.59) (Figure 4).

In the subgroup meta-analyses, a positive significant association was found between smoking and risk of extrahepatic CCA in studies performed in western countries (OR, 1.35; 95% CI, 1.17 to 1.55) and high-quality studies (OR, 1.29; 95% CI, 1.13 to 1.47). The association between smoking and risk of extrahepatic CCA was positive, but not significant in studies performed in Asian countries (OR, 1.29; 95% CI, 0.88 to 1.90) and low-quality studies (OR, 1.18; 95% CI, 0.63 to 2.22). When the meta-analysis was limited to studies that adjusted for potential confounders, the pooled data showed a positive significant association between smoking and risk of extrahepatic CCA in the studies that adjusted for diabetes mellitus (OR, 1.36; 95% CI, 1.16 to 1.58) and liver cirrhosis (OR, 1.35; 95% CI, 1.15 to 1.58), and a positive, but non-significant association was found in the studies that adjusted for cholelithiasis (OR, 1.26; 95% CI, 0.90 to 1.77).

### Sensitivity analysis and publication bias

In sensitivity analysis, each study was excluded and its influence was evaluated by calculating the pooled OR for the rest of the studies. The analysis confirmed the stability of the result because none of the individual studies markedly affected the pooled effect.

**Figure 5:**
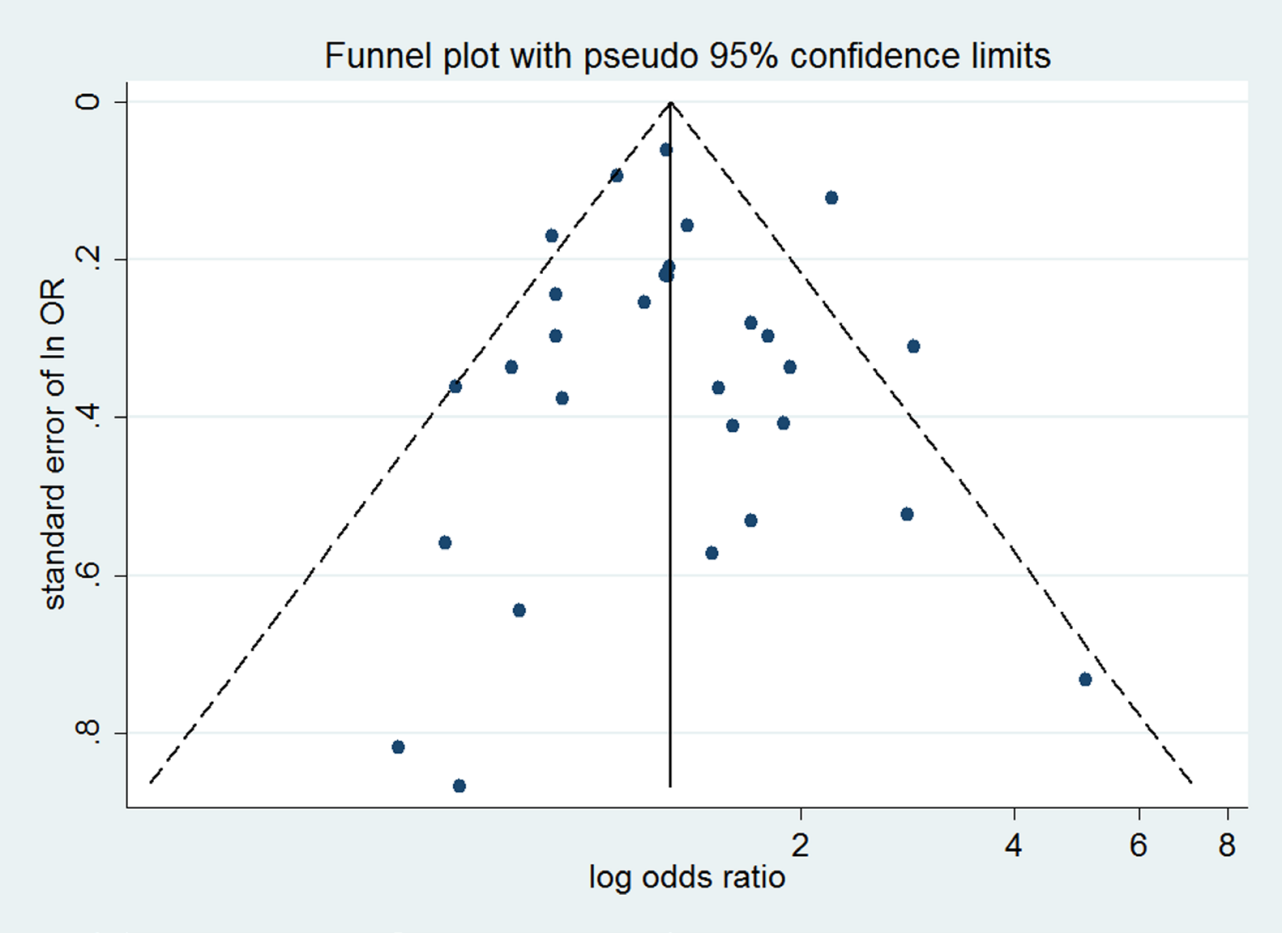
Funnel plot for assessing publication bias of smoking and risk of CCA

**Figure 6:**
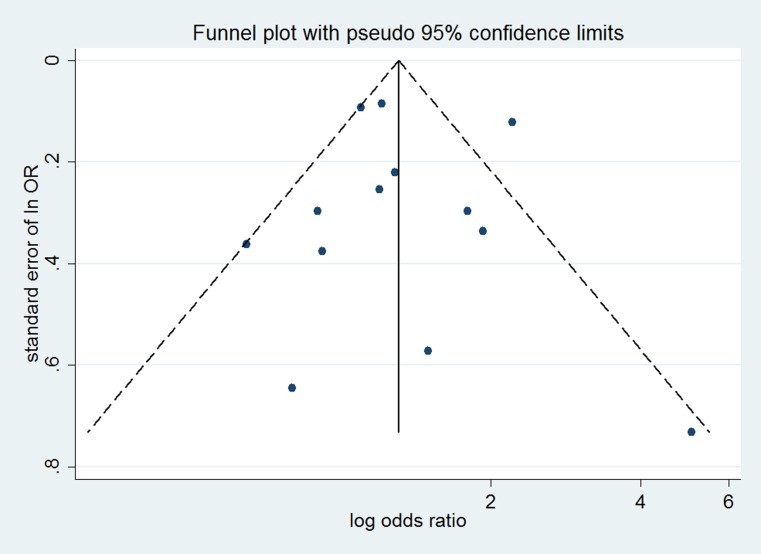
Funnel plot for assessing publication bias of smoking and risk of intrahepatic CCA

**Figure 7:**
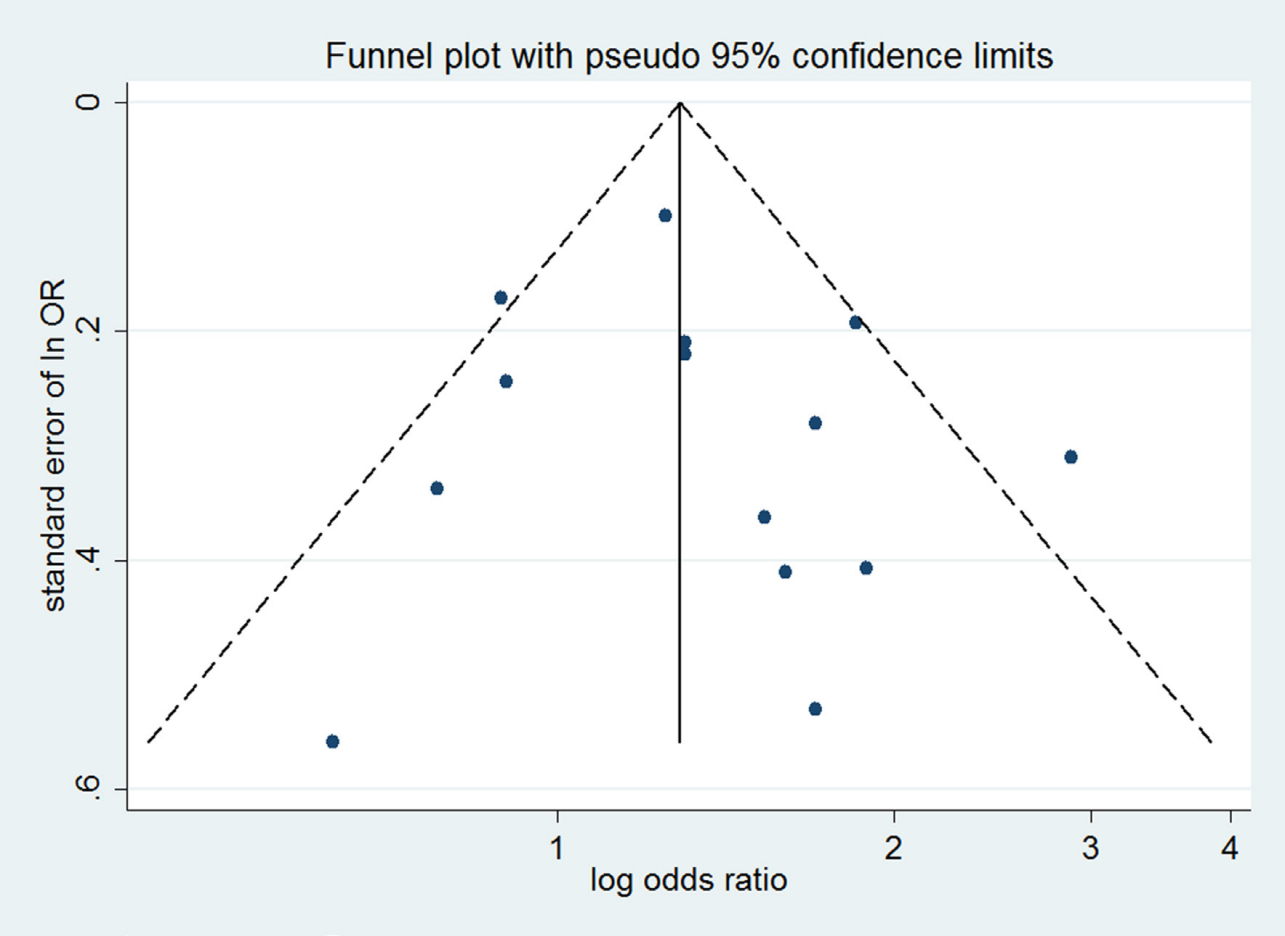
Funnel plot for assessing publication bias of smoking and risk of extrahepatic CCA

No evidence of significant publication bias was noted from visual inspection of the funnel plots (Figures 5–7), Begg’s test or Egger’s test for risk of CCA (Begg’s *P* = 0.626, Egger’s *P* = 0.954), risk of intrahepatic CCA (Begg’s *P* = 0.463, Egger’s *P* = 0.887), or extrahepatic CCA (Begg’s *P* = 0.584, Egger’s *P* = 0.564).

## DISCUSSION

The association between smoking and risk of CCA has been debated for a long time. By permitting a synthesis of data and providing an objective evaluation of the evidence, meta-analysis may be the resolution of controversy and uncertainty [50]. Our meta-analysis of 22 non-randomized studies demonstrated that smoking was associated with a modest but significantly increased risk of CCA. This modestly increased risk of CCA was consistently shown, irrespective of different study regions and discrepant quality of studies. Based on these findings, we believe that smoking increases the risk of CCA, although the effect is relatively small. This effect is much smaller compared with the associated risk in malignancies of the respiratory system and even lower compared with that of upper gastrointestinal carcinoma such as esophageal or gastric cancer [14, 51].

The pooled estimate from Asian populations was lower than that from Western populations. A similar difference was also observed in lung and bladder cancers. Gandini et al. [14] performed a meta-analysis to estimate the association between tobacco smoking and cancer risk, and their pooled data showed that the association of smoking with lung cancer in Asian populations was markedly lower than that in Caucasian populations. Van Osch et al. [52] performed a meta-analysis to assess the association between smoking and bladder cancer risk, and found that all studies in Asian populations showed lower ORs than pooled estimates from the United States and Europe. Why Asian populations present lower susceptibility for smoking-related cancers is still unclear. One explanation may be that nicotine intake from smoking is lower in Asian populations compared with that in Caucasian populations; thus Asian populations might be less susceptible to the harmful effects of cigarette smoking [53].

The results of our meta-analysis might be distorted by selection bias and confounding factors from non-randomized studies [54]. In general, a meta-analysis of randomized controlled trials (RCTs) allows for a more objective assessment of evidence than that of non-randomized studies. However, non-randomized studies should be retrieved for meta-analysis when RCTs are not available. To date, no RCTs have assessed the association between smoking and risk of CCA. Thus, this meta-analysis was conducted by pooling the findings from 22 non-randomized studies. Important risk factors of CCA, including diabetes mellitus, cholelithiasis, and liver cirrhosis, were carefully regarded as potential confounders in our analysis. The results showed that the association between smoking and risk of CCA remained unchanged after adjustment for these potential confounders. We could not evaluate any potential effect modification because the information on the effect modification between smoking and these CCA risk factors were limited.

Our results support that smoking is associated with increased risk of CCA, and such a relationship is further corroborated by biological plausibility. Several constituents in cigarette smoke have been shown to lead to the development of CCA in animal and human experiments. N-Nitrosodimethylamine, as mentioned in the introduction, can cause CCA in mice [13]. A strong relationship between vinyl chloride workers and CCA mortality has been reported [55]. Additionally, increased CCA risk due to arsenic in drinking water was also reported in the United States [56].

Our meta-analysis has some limitations. First, the adjustment factors were inconsistent across the studies. We attempted to employ the best adjusted estimate, which adjusted for as many potential confounders as possible. Second, all of the included studies were non-randomized studies owing to lack of relevant RCTs in the literature. Furthermore, one abstract without available full text was included in this meta-analysis, and the methodological quality was not assessed properly. Moreover, the information regarding the number of cigarettes smoked per day and duration of smoking was not provided in most studies, thus made evaluation of a dose–response trend difficult in this study.

In conclusion, the results of our meta-analysis support the hypothesis that there is a moderate association between cigarette smoking and risk of CCA. Further large-scale and well-conducted studies that investigate potential effect modification with confounders and the dose-response relationship between cigarette smoking and risk of CCA are needed. This conclusion delivers an important public health message to areas of both high CCA incidence and high smoking prevalence such as in China.

## MATERIALS AND METHODS

This systematic review and meta-analysis was performed in accordance with the Preferred Reporting Items for Systematic Reviews and Meta-Analyses statement [57].

### Search strategy

A systematic literature search of Embase, Pub Med, and Cochrane Central Register of Controlled Trials databases was performed to identify all relevant articles regarding the relationship between smoking and risk of CCA from inception to April 11, 2017. Both subject headings and free text words were used in the search. The following search terms were used: “smoking OR tobacco OR cigarette” AND “cholangiocarcinoma OR bile duct cancer”. The following trial registers were also searched to find potentially relevant ongoing trials: International Standard Randomised Controlled Trial Number registry, World Health Organization International Clinical Trials Registry Platform, and ClinicalTrials.gov. Additionally, we screened the reference lists from all retrieved articles for relevant studies. No language restriction was imposed in our search strategy.

### Eligibility criteria

Studies were included in this meta-analysis if they met the following criteria: (1) randomized controlled trials or non-randomized studies; (2) full-text articles and abstracts that included smoking as an exposure of interest; (3) the outcome of interest was CCA, intrahepatic CCA, extrahepatic CCA, perihilar CCA, or distal CCA; and (4) ORs or relative risk (RRs) with 95% CIs were reported or can be calculated. Studies were excluded if they were either of the following: (1) reviews, letters, editorials and case reports; (2) without data specific for CCA; (3) without appropriate data that could be extracted or calculated. In the case of multiple publications from the same population, only the most comprehensive one was included.

### Data extraction and quality assessment

The following data were collected from each study: publication data (i.e., the first author’s name, publication year, and study location), study design, study period, sample size, smoking status, OR (or RR) estimates with corresponding 95% CIs, and adjusting factors. When multiple estimates of effect (OR) were presented, the most adjusted one was extracted; when an adjusted estimate was not available, crude estimate was extracted. We attempted to contact the authors of relevant studies for important unreported data by e-mail communication. Data extraction was conducted independently by two reviewers, with disagreements resolved by discussion with a third reviewer.

Because all included studies were non-randomized studies, their methodological quality was assessed independently by three reviewers using the NOS [58]. The NOS evaluates the study quality based on three aspects: namely selection, comparability, and exposure (case-control studies) or outcome (cohort studies), using a star rating system ranging from zero to nine stars. Studies with seven or more stars were considered to be of high quality.

### Statistical analysis

Because the prevalence of cholangiocarcinoma is low, the RR mathematically approximates the OR [59]. The ORs with corresponding 95% CIs from individual eligible studies were combined to obtain a pooled OR with 95% CI. Statistical heterogeneity between studies was measured by using the Chi-square (χ^2^, or Chi^2^) test and quantified via I^2^ statistic; *P* value < 0.10 or I^2^ > 50% was considered statistically significant. When significant heterogeneity was observed, a random-effects model was used to calculate the pooled effect; otherwise, a fixed-effects model was applied. Once significant heterogeneity was found, we attempted to explore the causes of heterogeneity through a subgroup analysis by study location, study quality, and adjustment for confounders. To assess the stability of the results, we performed a sensitivity analysis by removing each study in the analysis at a time. Publication bias was estimated through both visual inspection of funnel plots and statistical evaluation with the Begg’s funnel plot and Egger’s tests [60, 61]. A two-sided *P* value less than 0.05 was considered statistically significant. All analyses were conducted using Stata version 12.0 software (Stata Corporation, College Station, TX).
